# Growth mechanism of strongly emitting CH_3_NH_3_PbBr_3_ perovskite nanocrystals with a tunable bandgap

**DOI:** 10.1038/s41467-017-00929-2

**Published:** 2017-10-17

**Authors:** He Huang, Johannes Raith, Stephen V. Kershaw, Sergii Kalytchuk, Ondrej Tomanec, Lihong Jing, Andrei S. Susha, Radek Zboril, Andrey L. Rogach

**Affiliations:** 1 Department of Materials Science and Engineering and Centre for Functional Photonics (CFP), City University of Hong Kong, 83 Tat Chee Avenue, Kowloon, Hong Kong SAR; 2 Regional Centre of Advanced Technologies and Materials, Faculty of Science, Department of Physical Chemistry, Palacky University Olomouc, Slechtitelu 27, Olomouc, 78371 Czech Republic; 3 Institute of Chemistry, Chinese Academy of Sciences, Bei Yi Jie 2, Zhong Guan Cun, Beijing, 100190 China

## Abstract

Metal halide perovskite nanocrystals are promising materials for a diverse range of applications, such as light-emitting devices and photodetectors. We demonstrate the bandgap tunability of strongly emitting CH_3_NH_3_PbBr_3_ nanocrystals synthesized at both room and elevated (60 °C) temperature through the variation of the precursor and ligand concentrations. We discuss in detail the role of two ligands, oleylamine and oleic acid, in terms of the coordination of the lead precursors and the nanocrystal surface. The growth mechanism of nanocrystals is elucidated by combining the experimental results with the principles of nucleation/growth models. The proposed formation mechanism of perovskite nanocrystals will be helpful for further studies in this field and can be used as a guide to improve the synthetic methods in the future.

## Abstract (web summary)

The development of perovskite nanocrystals is limited by poor mechanistic understanding of their growth. Here, the authors systematically study the ligand-assisted reprecipitation synthesis of CH_3_NH_3_PbBr_3_ nanocrystals, revealing the effect of precursor and ligand concentrations on bandgap tunability.

## Introduction

Metal halide perovskites of a general formula ABX_3_ (where A and B are monovalent and divalent cations, respectively, and X is a monovalent halide anion) are promising materials^1–7^ for a diverse range of applications such as light-emitting devices^8–20^, solar cells^21–26^, and photodetectors^27–31^. The first colloidal synthesis of the organic-inorganic CH_3_NH_3_PbBr_3_ perovskite nanocrystals (NCs) was reported in 2014 by Galian and Perez-Prieto, who used organic ammonium cations to stabilize small-sized crystallites in a suspension^32^; the same group later on enhanced their photoluminescence (PL) quantum yield (QY) to an impressive value of 82%^33^ and then 100%^34^. Soon after that first publication, Zhong’s group introduced a ligand-assisted reprecipitation (LARP) technique in a mixture of a good and a poor solvent to produce CH_3_NH_3_PbX_3_ (X=Cl, Br, I) NCs with a tunable bandgap by varying halide elements^35^. At nearly the same time, Kovalenko’s group introduced all-inorganic CsPbX_3_ perovskite NCs, which exhibited not only compositional (X=Cl, Br, I) bandgap engineering, but also the size-tunability of the bandgap depending on the reaction temperature^36^. In our previous related report^37^, we demonstrated the bandgap tunability of CH_3_NH_3_PbBr_3_ NCs by changing the temperature of the poor solvent to exert control over the LARP process, and achieved PL QY of 93%. The coating of perovskites has proven to be an efficient way to enhance their stability^13, 38–41^. Several techniques have recently been introduced for the room temperature (RT) growth of perovskite NCs: emulsion-based synthesis^42^; modified reprecipitation method for the CsPbX_3_ system^9^; synthesis of CsPbBr_3_ nanoplates using an adaptation of Galian/Perez-Prieto’s method^43^; reprecipitation strategy leading to different shapes of the final products (spherical dots, nanocubes, nanorods, and nanoplates)^44^; the employment of branched capping ligands^45^; the in-situ formation of CH_3_NH_3_PbBr_3_ NCs in polymer matrix^46^; and top-down fabrication by employing ligands as coordinating solvents^47^. The formation of different shapes of perovskite nanoparticles has been demonstrated as well^43, 48–55^.

Metal halide perovskites have rather low formation energy and fast crystallization rate; owing to the fast nucleation and growth of the perovskite NCs in most of these methods, it is hard to address their formation mechanism. De Mello and Kovalenko used a microfluidic flow reactor platform to shed light on the formation process of CsPbX_3_ NCs and to optimize the synthesis parameters^56^. Snaith’s group focused on the formation of bulk perovskite crystals with respect to their supersaturation and subsequent crystallization^57^. Pan et al.^58^ discussed the influence of different ligands on the growth of CsPbBr_3_ NCs. There remains a clear lack of a systematic study on the combination of factors governing the wet chemical synthesis of CH_3_NH_3_PbBr_3_ perovskite NCs with a tunable bandgap, and the related growth mechanism. Such a study would trigger further developments in the colloidal perovskite NC field, and provide a guidance to improve the existing synthetic methods.

In this work, we demonstrate how the LARP procedure leads to the bandgap tunability of CH_3_NH_3_PbBr_3_ NCs synthesized at both RT and elevated (60 °C) temperature through the variation of two important parameters governing colloidal NP growth: the precursor and the ligand concentrations. We discuss in detail the role of two coordinating ligands, oleylamine (OLA) and oleic acid (OA), which are commonly used for the synthesis of perovskite NCs, and propose a qualitative model for the perovskite NC nucleation and growth.

## Results

### Fabrication of CH_3_NH_3_PbBr_3_ NCs

The LARP^19, 37^ synthesis of CH_3_NH_3_PbBr_3_ NCs was carried out as follows: 0.5 mL aliquots of a good solvent (*N*-dimethylformamide, DMF) containing variable amounts of perovskite precursors (PbBr_2_ and CH_3_NH_3_Br) and a fixed amount of two ligands (5 μL OLA and 50 μL OA) were quickly injected into 5 mL volumes of toluene as a poor solvent under vigorous stirring, which was either kept at RT (20 °C) or was pre-heated to 60 °C in an oil bath. We define certain concentrations of the precursors (0.02 mmol PbBr_2_ and 0.016 mmol CH_3_NH_3_Br) as the standard concentration set (1×)^37^. Other synthesis batches reported in this study were carried out at proportionally decreased (or increased) concentrations of these two precursors as referred to the standard one. For example, for the sample labeled 0.33×, the amount of precursors was 0.0066 mmol PbBr_2_ and 0.0053 mmol CH_3_NH_3_Br. In another set of syntheses, 0.5 mL of DMF aliquots containing a fixed amount of perovskite precursors (1×) and variable amounts of the two ligands were injected into 5 mL volumes of toluene at RT. The amounts of OLA and OA varied as 2.5/25 μL; 3.8/38 μL; 5/50 μL and 10/100 μL, respectively.

**Fig. 1:**
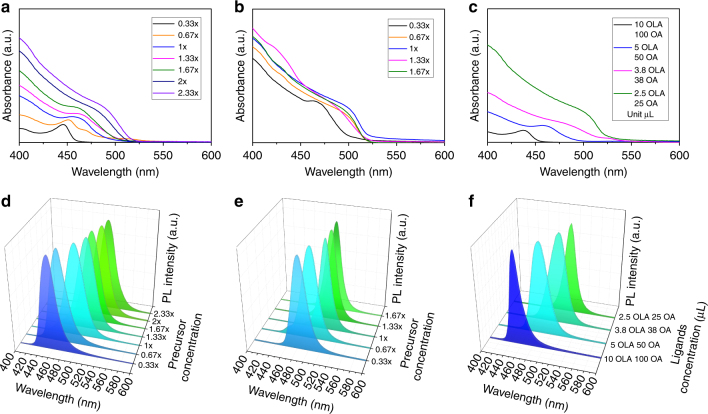
Optical absorption and photoluminescence spectra of the samples. Optical absorption (**a**, **b**, **c**) and photoluminescence (PL) spectra (**d**, **e**, **f**) of CH_3_NH_3_PbBr_3_ NCs. Samples were synthesized at room temperature (RT) (**a**, **d**) and at 60 ^o^C (**b**, **e**) with a varying amount of precursors and a fixed amount of ligands (5 μL oleylamine—OLA/50 μL oleic acid—OA). **c**, **f** show the spectra for the RT synthesis with the fixed amount of precursors (1×) but with varying amount of ligands

Color change from light yellow to yellow-green is observed immediately after injection, indicating the formation of perovskite NCs, which in most cases is also accompanied by the formation of some amount of larger agglomerates; they can be separated from the clear supernatant NC solution either by naturally allowing them to precipitate, or by centrifugation. Optical absorption and PL spectra of CH_3_NH_3_PbBr_3_ NCs gradually shift toward longer wavelengths upon increasing the concentration of the precursors, while leaving the amount of the ligands constant (5 μL of OLA and 50 μL of OA). Figure 1a, d demonstrates this tunability for the synthesis carried out at RT. The absorption peak position ranges from 446 to 488 nm (Fig. 1a), and the PL maxima cover the range of 455 to 516 nm (Fig. 1d), corresponding to change in emission color from blue to green. For the synthesis performed at 60 °C (temperature of the poor solvent toluene), the PL maximum of the NCs shifts from 483 to 512 nm as the concentration of the precursors increased (Fig. 1e). At this higher reaction temperature, the formation of perovskite NCs accelerates and the saturation of the PL peak position occurs more rapidly. For relative concentrations of the precursors of 1×, 1.33×, and 1.67×, the perovskite NCs did not grow anymore (i.e., the absorption maxima (Fig. 1b) and PL spectra peaks (Fig. 1e) did not red-shift), while mostly the amount of precipitate increases, which will be addressed in detail later on when we discuss the growth mechanism.

Figure 1c, f shows absorption and PL spectra for the perovskite NCs synthesized at the constant precursor concentration (1×) while raising the concentration of the ligands. The PL emission maxima of the NCs blue-shifted to shorter wavelength from 513 to 452 nm when the amount of ligands was increased from 2.5 μL OLA/25 μL OA to 10 μL OLA/100 μL OA (Fig. 1f). This implies that the higher amount of ligands leads to the formation of smaller perovskite NCs, with a higher degree of quantum confinement (the Bohr radius of CH_3_NH_3_PbBr_3_ is 2 nm^59^). We note that similar kinds of ligands have been found to be able to break bulk perovskites into small light-emissive particles^60^.

### Characterization of CH_3_NH_3_PbBr_3_ NCs

**Fig. 2:**
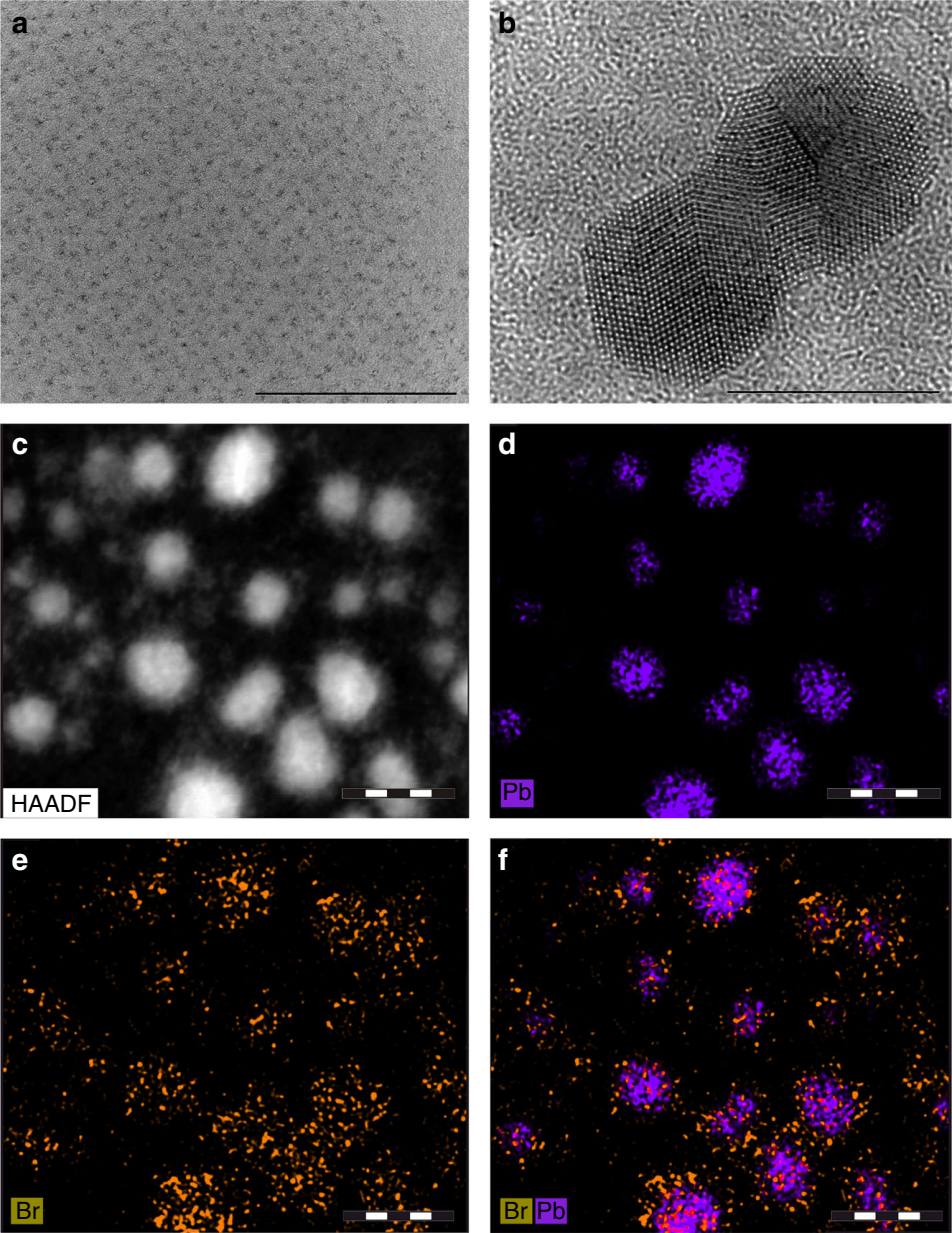
Structural and compositional analysis of CH_3_NH_3_PbBr_3_ NCs. **a** TEM *overview image* of CH_3_NH_3_PbBr_3_ NCs (*scale bar*, 100 nm) and **b** HRTEM image of selected larger CH_3_NH_3_PbBr_3_ NCs (1×) synthesized at room temperature (*scale bar*, 10 nm). **c** HAADF image and **d**–**f** corresponding elemental mapping of Pb and Br in CH_3_NH_3_PbBr_3_ NCs (*scale bar*, 20 nm)

Figure 2 shows transmission electron microscopy (TEM) and high-resolution TEM (HRTEM) images of a representative sample (1×) of the CH_3_NH_3_PbBr_3_ NCs synthesized at RT. The NCs are fairly monodisperse (Fig. 2a), but with some presence of larger outlier particles such as presented in the HRTEM image of Fig. 2b to demonstrate their highly crystalline structure. We calculated sizes from the TEM images (Supplementary Fig. 1), the average size of 0.33×, 1×, 2× precursor concentration samples was 2.2, 2.9, and 3.7 nm, respectively. Size distribution histograms (each obtained on 200 particles) are given in Supplementary Fig. 1d, e, and f, respectively. The gradually increased sizes further confirm the size effect of the as-prepared perovskite NCs and are consistent with the red-shift of PL peaks with the increase of precursor concentration. Combination of the high-angle annular dark-field imaging (HAADF) with an energy-dispersive X-ray elemental mapping confirm the presence of the constituent elements of the perovskite NCs, namely Pb and Br (Fig. 2c–f). Energy-dispersive spectra additionally confirmed the presence of N originating from the organic ammonium cation. Energy-dispersive X-ray spectroscopy (EDS) spectra (Supplementary Fig. 2) show the presence of Pb and Br in all the samples even for the limited contents in sample 0.33×. The relative ratio of Pb/Br increases in the order of 2×, 1×, 0.33×; this tendency may be caused by more coordination of the ligands to surface Pb^2+^ in smaller NCs leading to the relative lack of the detected Br element.

We also performed X-ray diffraction (XRD) measurements to further confirm that all the samples were perovskite in structure and there were no changes in the crystalline phase when changing the synthesis parameters. The XRD patterns of the powdered NCs (Supplementary Fig. 3) retain the peaks corresponding to CH_3_NH_3_PbBr_3_ perovskite precipitate, which are superimposed on a broader background peak originating from organic ligands, as has been assigned in a previous report^50^. The full width at half maximum of the diffraction peaks becomes broader for smaller NCs, and the crystallinity of the samples decreases when moving from the bulk precipitate toward smaller sized NC samples.

To reveal the nature of the interaction between the perovskite species at the NC surface and the actual ligands, we performed X-ray photoelectron spectroscopy (XPS) and Fourier transform infrared spectroscopy (FTIR) measurements. XPS survey scans of perovskite NCs prepared with precursor concentrations of 0.33× (a), 1× (b), 2× (c), and of the 2× precipitate (d) with the relative quantifications are shown in Supplementary Fig. 4. High-resolution XPS spectra were measurable only for samples 2× and 1× due to the reasonable Pb contents. The sample 0.33× showed very low Pb content in XPS, but we confirmed the presence of Pb and Br by EDS spectra, as mentioned above. The XPS spectrum of Pb 4 f in Supplementary Fig. 5 shows two symmetric peaks attributed to Pb 4f_7/2_ and Pb 4f_5/2_ levels. The small peak at a lower-binding energy could be attributed to the presence of metallic lead^33^. Due to a limited amount of Pb, the partial reduction/oxidation during the sample pretreatment may also lead to the formation of Pb or PbO. The Br 3d peaks shown in Supplementary Fig. 6 can be fitted into two peaks centered at 69 and 70.3 eV, corresponding to the inner and surface Br^−^ ions, respectively. The N 1 s spectrum shown in Supplementary Fig. 7 has two peaks, implying the two existing chemical states of the N element. The peak at 398.6 eV can be attributed to the presence of OLA, while peak at 400 eV originates from methylamine salt. The O 1 s XPS spectrum as shown in Supplementary Fig. 8 also contains two peaks. The lower energy peak at 532.3 eV results from two nonequivalent O atoms of carboxylic acid, while the higher energy state peaking around 533.7 eV can be assigned to two chemically equivalent O atoms from carboxylate species of deprotonated oleic acid.

Before moving to the interpretation of FTIR data obtained on the perovskite NCs of lower to higher precursors concentration (0.33×, 1×, 2×), 1× and 2× precipitates, as well as OL and OLA ligands and the CH_3_NH_3_Br precursor as shown in Supplementary Fig. 9, we would like to stress that we do not expect that different concentration of the precursors would lead to the different coordination of ligands to Pb^2+^. In our view, the FTIR data may be especially useful to prove the coordination of Pb and OA when we perform the normalization according to the most pronounced absorbance of the latter at 2925 cm^−1^, as shown in Supplementary Fig. 10. Compared to pure OA, the proportions of C=O stretch band of free carboxyl group (peaking at 1722 cm^−1^) for three NCs samples (0.33×, 1×, 2×) are obviously decreased. This arises from the transformation from two nonequivalent oxygen atoms in R-COOH toward two chemically equivalent oxygens in R-COO^−^, with the later one bearing delocalized electron from carboxylate group. This indicates that OA forms a primary amine salt with OLA by donating its proton, leading to the formation of oleate, which then coordinates with Pb by partly replacing the bromide ion of PbBr_2_ by the carboxylate group, because the binding constant of the carboxylate group with Pb^2+^ (lg *k*_1_ = 2.52) is quite comparable with the second step binding constant (lg *k*_2_ = 0.7) for PbBr_2_^61^. Even if we exclude the contribution of OLA (ratio of OLA to OA in precursors is 1:10) to saturated vibration of C–H (2925 cm^−1^), the proportions of C=O stretch band of free carboxyl group decreased approximately by 15–36% calculated by integration. Furthermore, according to hard and soft acids and bases theory, Pb^2+^ is a borderline acid, whereas deprotonated OA and OLA are both hard bases. Besides deprotonated OA, OLA as a hard base can also readily coordinate with Pb. It has been previously demonstrated that OLA is key to control the crystallization of CH_3_NH_3_PbBr_3_ NCs^35^.

**Fig. 3:**
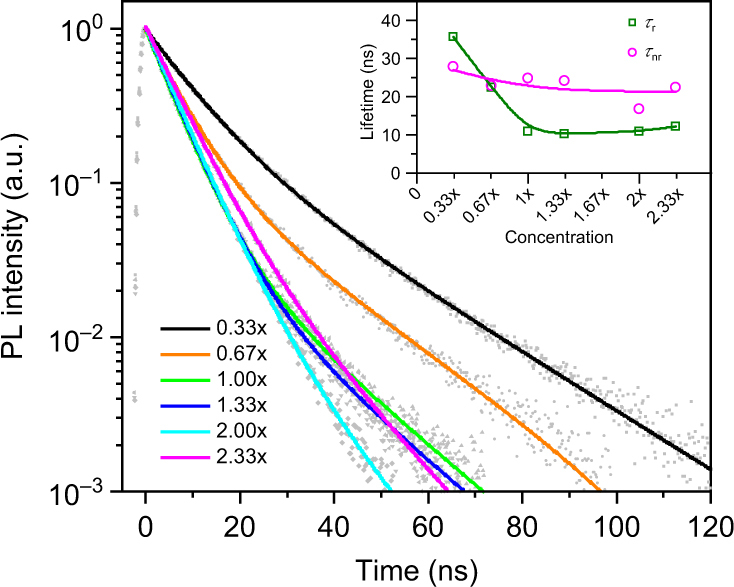
Time-resolved photoluminescence decays of the samples. Time-resolved photoluminescence (PL) decays (*symbols*) and their biexponential fits (*solid lines*) for CH_3_NH_3_PbBr_3_ NCs synthesized at room temperature are shown for different relative concentrations of the precursors, as indicated. *Inset* illustrates the trends of radiative (*τ*_r_) and nonradiative (*τ*_nr_) recombination lifetimes as a function of the relative precursor concentration; connecting lines are just a guide for the eye

The PL QY of the CH_3_NH_3_PbBr_3_ NCs was measured by an absolute method using a fluorescence spectrometer equipped with an integrating sphere, with excitation at 405 nm. The PL QY for the series of CH_3_NH_3_PbBr_3_ NCs synthesized at RT with a variable concentration of precursors started from 44% for the sample 0.33× and gradually increased to 65% for the highest concentration used (2.33×). Time-resolved PL decays of these CH_3_NH_3_PbBr_3_ NCs measured at the same excitation wavelength of 405 nm are presented in Fig. 3. All PL decays were fitted to a biexponential decay function and average PL lifetimes in the range of 5.9–15.6 ns were extracted. From the average PL lifetimes and PL QY values, we calculated the radiative (*τ*_r_) and nonradiative (*τ*_nr_) lifetimes of perovskite NCs, which are given in the inset of Fig. 3 as a function of the relative precursor concentration (ranging from 0.33× to 2.33×). Radiative lifetime decreases from 35 to 10 ns when the relative concentration of the precursors increases from 0.33× to 1×, and remains at this value for all the higher concentrations. The nonradiative lifetime fluctuates between 28–12 ns and remains slower than the corresponding radiative lifetime for the relative precursor concentration 1× and higher, resulting in the higher PL QY of the larger CH_3_NH_3_PbBr_3_ NCs.

From XPS data, regarding to the interaction of NCs and ligands, the capping ligands will be mostly coordinating at Pb sites, in analogy to the same ligands coordinating to metal cations on the surface of classical quantum dots such as CdSe or PbSe. There is a large body of literature existing on ligand coordination in those NCs, including nuclear magnetic resonance (NMR) studies^62–64^. There are also recent NMR studies on lead-based perovskite NC surface chemistry showing different coordination forms of Pb and ligands^65^. Via ^1^H solution NMR spectroscopy with 2D NOESY (nuclear Overhauser effect spectroscopy), Kovalenko and Hens found out that OLA on one hand is involved in the acid-base equilibrium with hydrogen bromide, and binds to the surface as oleylammonium bromide in the particle stabilization (binding to halide sites), which leads to highly dynamic character of ligand binding^65^. Ligands can be defined as L- and X-type, depending on the number of electrons that the neutral ligand contributes to the metal–ligand bond (either 2 or 1). Various (cation rich) metal sulfide and selenide NCs, including for example CdSe, CdTe, PbS, and PbSe, proved to be coordinated by X-type ligands such as carboxylates or phosphonates that bind to excess surface cations. L-type ligands are Lewis bases that in the case of binary NCs will coordinate to acidic surface cations. On the other hand, OLA also helps to deprotonate the OA to form oleylammonium oleate, and moreover the authors infer that oleate is binding to the NC surface (cation sites) as an ion pair with oleylammonium. This is a pair of X-type ligands, coordinating to a stoichiometric surface. Besides, OLA could also bind in its unprotonated state, as an L-type ligand coordinating to the surface cations. Furthermore, the resulting oleylammonium oleate presents high-binding affinity to particle surface and as a consequence leads to both colloidal and PL stability. The presence of an amine excess in the solution may cause the occurrence of a strongly bound fraction of OA and results in high PL QYs. It is noted that we employed a longer carbon chain amine than in the previous report of Zhang et al.^35^. On the basis of this and the above studies, we focus on the interaction of the ligands with surface Pb atoms for next.

## Discussion

With respect to involvement of OLA and OA as a capping ligand pair, their collective effort and their ratio to Pb^2+^ regulates the chemical environment of the Pb precursor and surface Pb sites. They play important roles in adjusting the reactivity of the precursor and controlling the access rate of precursors to the NC surface and consequently regulate the size of the resultant particles. To access the surface coordination of the perovskite NCs by ligands, the proportion of surface Pb atoms was estimated for different sizes of NCs; the details are given under Methods. In the NC size range of 2–5 nm, the number of surface lead atoms per particle increases from 36 to 223 atoms. At 5 nm size, 71% of the Pb atoms are in surface accessible sites and so potentially require a matching proportion of ligands. However, in all the different syntheses we carried out, the combined total molar amounts of OLA and OA were always in excess of the number of surface Pb sites.

Interactions among Pb^2+^, OA, and OLA may occur in the following three ways. (i) OLA as a coordinating agent can directly bind to Pb^2+^, as it can readily donate its lone-pair electrons on the N atom to Pb^2+^ to form a Pb^2+^–oleylamine complex. (ii) OA readily reacts with OLA by donating its proton, forming a primary amine salt, and then the deprotonated OA can directly coordinate with Pb^2+^ or replace the bromide ions of PbBr_2_. It is also worth mentioning here that Pb^2+^ could in principle exhibit coordination numbers in the range between 2 and 10^66^, therefore, there is a great possibility for depronated OA to directly bind Pb^2+^ without any need to replace the existing bromide ions of PbBr_2_. (iii) Considering that OA was mixed with OLA at a molar ratio (10:1) much higher than stoichiometric primary amine salt, Pb–oleate coordination can also occur upon the substitution of the bromide ion of PbBr_2_ by the oleate group without the help of the OLA, because the binding constant of the carboxylate group with Pb^2+^ (lg *k*_1_ = 2.52) is quite comparable with the second step binding constant (lg *k*_2_ = 0.7) for PbBr_2_^61^. Although OA may replace the bromide ion of PbBr_2_ to form Pb-oleate, the kinetics is slower in comparison with kinetically favorable coordination of OLA to Pb^2+^. The chemical forms of the Pb precursor in the presence of the two capping ligands can be affected and become more complicated upon imposing a pre-aging process or adjusting the ligand feeding sequence. In fact, it was previously demonstrated that the chemical forms of the metal-organic precursors are directly correlated with the effective supplying rate of the precursors when forming target NCs, consequently exhibiting strong influences on the size-regulation ability^67, 68^.

**Fig. 4:**
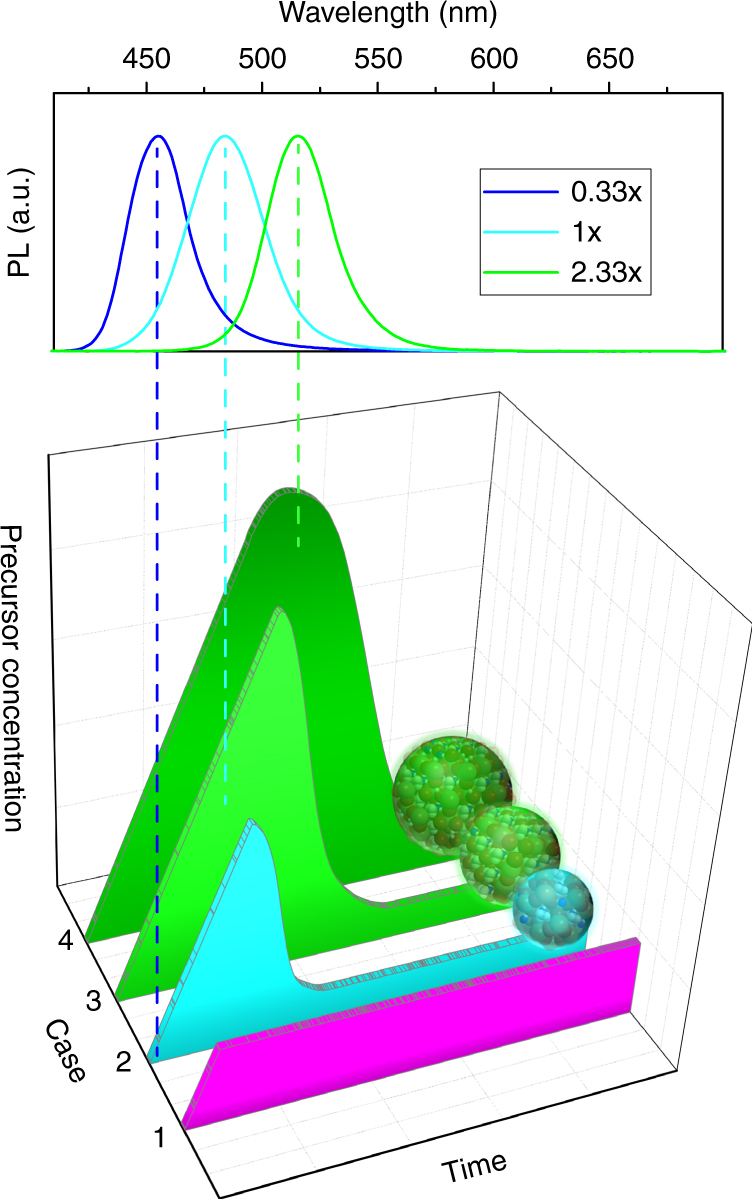
Formation process model adopted for the discussion of perovskite nanocrystal (NC) growth: Variation of the precursor concentration with time (a.u.) for four different cases (1–4). *Upper frame* shows positions of PL spectra for the perovskite NCs characteristic for certain discussed cases, indicated by *vertical dashed lines*. In the first case, the concentration of the precursors is too low (0.01×) for any nucleation process. In the second case, the precursors’ concentration (0.33×) is not yet high enough to allow for much growth of the NCs after nucleation, but will be sufficient for the formation of small NCs. In the third case, with more precursors available in the reaction mixture (1×), the probability of the formation of larger perovskite micro-particles becomes higher. There are two competing processes: nucleation with and without further growth, while the formation of larger NCs for higher precursor concentrations is more likely to happen, and the amount of the non-NC precipitate is increased. In the fourth case, at the highest employed precursor concentration (2.33×), the nucleation and growth always happen until the excess concentration of the precursor falls to a low enough level

We now turn to the discussion of the qualitative model for the perovskite NC growth. Often particle nucleation and growth mechanisms are discussed in terms of LaMer and Dinegar’s model^69^ for the formation of classic colloids from a single monomer precursor. CH_3_NH_3_PbBr_3_ NCs studied in this work are synthesized by the LARP approach^35^, where the perovskite nano- (and/or micro-) particles are formed from precursors in a very short period of time (within few seconds) as a result of the supersaturation of all of the precursors induced by the mixing of the good and the poor solvent. The concentration of precursors and the amount of ligands in the good solvent determine the formation process of perovskite NCs. Figure 4 shows four different cases for our nucleation and growth model which we have adopted to discuss the experimentally observed trends on our perovskite system, linked to the position of PL spectra, which are also presented in Fig. 4 for the three representative cases. Our model describes the bandgap-tunability of NCs upon the change of concentration of the precursors. In contrast to the classic LaMer model where the nucleation occurs in one solvent with one precursor, the technique employed here contains two different solutions, two precursors and ligands, and the nucleation happens after the two solvents are rapidly mixed. In our four case model presented in Fig. 4, at the initial stage, a 0.01× concentration batch was used for synthesizing one sample. For this sample, no PL emission was observed even if the reaction mixture was kept for a long time. The precursors at this low concentration will be soluble in the good/poor solvent mixture without formation of any NCs. In this first initial case, the concentration of the precursors is thus too low for any nucleation process. In the second case, with the increase of the precursor concentration (samples 0.33× and 0.67×), NCs do form and the PL peak of the NCs is in the blue with only a little shift to the red during growth (Fig. 4, *upper part*) and no precipitate of larger bulk-like particles and aggregates is formed. The surface quality of the NCs is relatively low as their PL QYs remains below 50%. In this second case, the precursors’ concentration is not yet high enough to allow for much growth of the NCs after nucleation, but will be sufficient for the formation of small NCs.

In the third case, once the concentration reaches a certain level (samples 1× to 2×), there is clear evidence for the gradual and rather significant red-shift of the PL peak (Fig. 1a), which is accompanied by the shortening of the radiative lifetimes (compared to samples 0.33× to 1×) as demonstrated in Fig. 3. The decrease of radiative lifetimes when the emission shifts to longer wavelengths and also the increase of PL QY toward 70% may be linked to a change in the NC stoichiometry as their size increases. At the same time, with more precursors available in the reaction mixture, the probability of the formation of larger perovskite micro-particles becomes higher. We observed an increased amount of the precipitate, e.g., 60 mg per batch for the sample 2× vs. to 30 mg for the sample 1×. In the third case, we thus have two competing processes: nucleation with and without further growth, while the formation of larger NCs for higher precursor concentrations is more likely to happen, and the amount of the non-NC precipitate is increased. For the RT synthesis (Fig. 1a) the emission peak position shifts slower at higher concentration, but there is still a clear difference between samples 2× and 2.33×. For the higher reaction temperature, the growth process is accelerated, which is confirmed by the PL trends for the synthesis at 60 °C (Fig. 1b). From the thermodynamics aspect, the elevated temperature may lead to the increased solubility of the precursors in the reaction system, reducing the amount of precursors consumed during the nucleation process due to the reduced supersaturation degree, which is consequently favorable for growing larger particles out of the reaction system. It is worth mentioning that the increased ratio of Pb:ligand may also help to activate the particle surface by decreasing the surface density of the capping ligand, increasing the surface accessibility for the precursors and yielding a higher growth rate. The emission peak position at the same precursor concentration for the reaction at 60 °C will be red shifted compared to the sample synthesized at RT, while at the higher precursor concentration (i.e., 1×–1.67×) there will be almost no shift in the peak position, because at this higher temperature the reaction is accelerated thus making the concentration reach the maximum PL of NCs achieved at a lower concentration than at RT.

In the fourth case, at the highest employed precursor concentration of 2.33×, the NC solution is not stable and the precipitate has a tendency to form instantaneously, even when we remove it by centrifugation. In that case, the nucleation and growth always happen until the excess concentration of the precursor falls to a low enough level. The particles formed may be not entirely protected by ligands, which also decreases the NC’s colloidal stability.

The tendencies that we observe when we vary the amount of ligands (Fig. 2c) could be explained by this model as well. At the higher concentration of ligands, the NC growth becomes slow, resulting from the decreased reactivity of the precursors and a relatively inert, well passivated particle surface as well. The precursor concentration needed to reach to the same PL peak position has to be increased in the case of higher ligand concentrations. If we keep the concentration of the precursor constant, while changing the concentration of ligands, the PL peak position will be blue-shifted, as at the higher ligands concentration, the situation will be similar to the case of lower precursor concentration. Even though larger perovskite micro-particles may be formed, the large excess of ligands would have the ability to render them down into smaller NPs^60^ or suppress their formation in the first place.

In summary, we have shown that the LARP technique is a powerful method to synthesize bandgap-tunable CH_3_NH_3_PbBr_3_ NCs both at RT and at elevated temperature (60 °C) by using different concentrations of the precursors. The emission peaks of the synthesized NCs covered the range of 455–516 nm with narrow emission line widths of 29–41 nm and high absolute PL QYs reaching 70% for larger particles. We discuss in details the role of the two ligands (OA and OLA), which are commonly used for the synthesis of CH_3_NH_3_PbBr_3_ NCs in terms of the coordination of the lead precursors and the NC surface. Chemical forms of the Pb precursor in the presence of these two capping ligands are affected upon imposing a pre-aging process and/or adjusting the ligand feeding sequence. Chemical forms of the metal-organic precursor are directly correlated with the effective supplying rate of the precursors when forming target NPs, which consequently exhibits strong influences on the size-regulation ability. The growth mechanism of perovskite NCs is elucidated by combining the experimental results with the principles of nucleation/growth models. The proposed formation mechanism of perovskite NCs will be helpful for further studies in this field and will be used as a guide to improve the synthetic methods.

## Methods

### Materials

All reagents were directly used as received: PbBr_2_ (99.999%, Aldrich), methylammonium bromide (Dyesol), oleylamine (OLA, 98%, Aldrich), oleic acid (OA, 90%, Aldrich), DMF (99%, Sigma-Aldrich), toluene (99.7% GC, Sigma-Aldrich).

### Fabrication of CH_3_NH_3_PbBr_3_ NCs

In a typical synthesis of CH_3_NH_3_PbBr_3_ NCs, 0.5 mL *N*-dimethylformamide (DMF) containing variable amounts of perovskite precursors (we refer to a concentration of 0.02 mmol PbBr_2_ and 0.016 mmol CH_3_NH_3_Br as 1×) and a fixed amount of two ligands (5 μL OLA and 50 μL OA) was quickly injected into 5 mL of toluene as a poor solvent under vigorous stirring. The latter was either kept at RT (20 °C) or was pre-heated to 60 °C in an oil bath. The reaction time in all cases was within a few seconds. We defined concentrations of the precursors PbBr_2_ and CH_3_NH_3_Br used in our previous report^37^ (0.02 mmol PbBr_2_ and 0.016 mmol CH_3_NH_3_Br) as the standard concentration set (1×). Other syntheses reported in this study were carried out at proportionally decreased (or increased) concentrations of these two precursors as referred to this standard one. On the fixed perovskite precursor concentration, different amount of the two ligands were used to carry out similar reactions as above.

### Characterization

TEM imaging was carried out on a Philips CM-20 and TEM measurements with related elemental analysis were performed on a TITAN microscope with an X-FEG type emission gun, operating at 60–300 kV, and equipped with a Cs image corrector and a STEM HAADF, providing STEM-EDS. Powder XRD patterns were taken on a Philips X'Pert X-ray diffractometer using Cu Kα radiation (*λ* = 1.5418 Å). XPS measurements were performed on an ESCALAB-MKII 250 photoelectron spectrometer (Thermo, USA). FTIR measurements were done on a Perkin Elmer 16PC FT-IR spectrophotometer. Absorption spectra were obtained on a Cary 50 ultraviolet–visible spectrophotometer (Varian). PL spectra were measured on a Cary Eclipse (Varian) model and also a FLS920P fluorescence spectrometer (Edinburgh Instruments) equipped with a photon counting photomultiplier (R928P, Hamamatsu), with a 450 W xenon arc lamp as the excitation source for steady state and integrated QY measurements. The PL QY, defined as the ratio between photons emitted and absorbed by the sample, was determined by an absolute method using an integrating sphere with its inner face coated with BENFLEC®, fitted to the spectro-fluorimeter. The average PL decay lifetimes were measured using a 405 nm, 49 ps pulse width laser and a time correlated single photon counting system. Decay curves were fitted to multiple-exponential decay curves and the average lifetimes were calculated as, ${\tau _{{\rm{avg}}}}{\rm{ = }}{\sum} {{B_i}} \tau _i^2{\rm{/}}{\sum} {{B_i}{\tau _i}}$, where *B*_i_ are the amplitudes of the component decay times *τ*_i_.

### Surface lead calculation

The structure of CH_3_NH_3_PbBr_3_ is cubic with a lattice parameter, *a*, of 0.5933 nm. The volume of each unit cell is 0.2088 nm^3^, and the surface area of a single unit cell face is 0.3520 nm^2^. If we assume the nanoparticle (NP) is approximately a sphere then when the NP diameter is 2 nm, the volume of the NP is 4.1888 nm^3^, which is equivalent to 20 unit cell volumes. The surface area of a 2 nm NP is 12.6 nm^2^, which is equivalent to 36 unit cell faces each of area *a* ^2^. This implies that up to 16 of the 20 unit cells that make up the NP volume are showing 2 or more faces at the surface. Unit cells that are fully buried within a NP (no faces at the surface) will contribute exactly the standard perovskite stoichiometry per unit cell, i.e., each contains 1 Pb, 1 methylammonium, and 3 halide ions per unit cell. However, when a unit cell is at the surface (assuming each unit cell is complete), the stoichiometry is no longer 1:1:3. If we assume the Pb rich unit cell surface, then a unit cell presenting one face at the NP surface contains 1.5 Pb ions per unit cell volume, with the exposed face accounting for 1 Pb ion. For a unit cell with two exposed surfaces there are 2.25 Pb ions per unit cell volume, with 1Pb per exposed unit cell face. Thus, for the 2 nm diameter NPs it can be estimated that up to 66 Pb atoms are contained in the volume, with up to 36 of them located on the NP surface, or almost 55% of the Pb atoms are surface accessible.

### Data availability

All data generated or analyzed during this study are included in this published article (and its Supplementary Information files). All relevant data are available from the authors.

## Electronic supplementary material

Supplementary Information
