# Supplementary material for: Growth mechanism of strongly emitting CH3NH3PbBr3 perovskite nanocrystals with a tunable bandgap
Source: Nat Commun. 2017 Oct 17;8:996. doi: 10.1038/s41467-017-00929-2 (PMC5715004; doi:10.1038/s41467-017-00929-2)
Supplement: Supplementary file 1 — Supplementary Information [file 41467_2017_929_MOESM1_ESM.pdf]

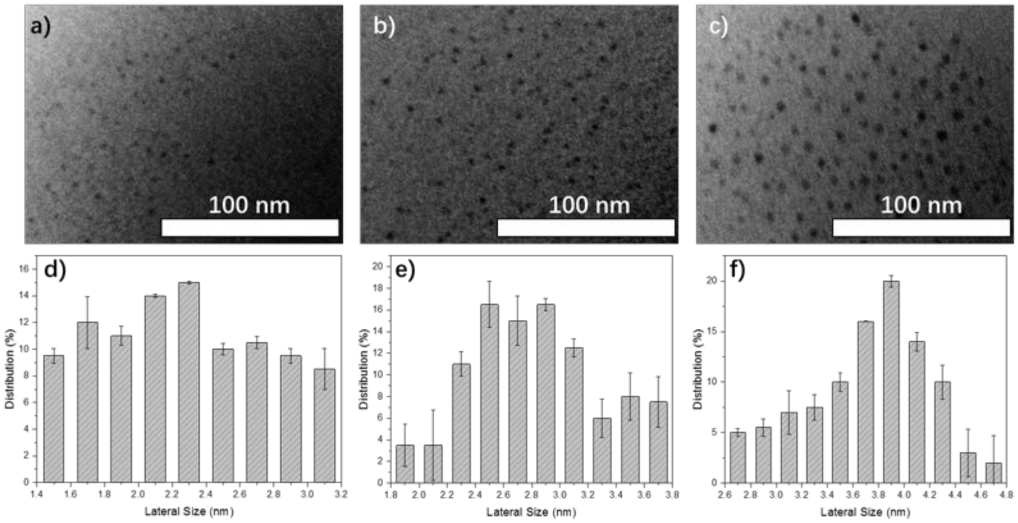

Supplementary Figure 1 TEM images of perovskite nanocrystals prepared with the precursor concentration of a) 0.33x, b) 1x, and c) 2x, respectively. Size distribution histograms with standard deviation error bar of Gaussian fitting for the three samples are shown for samples d) 0.33x, e) 1x, and f) 2x, respectively.

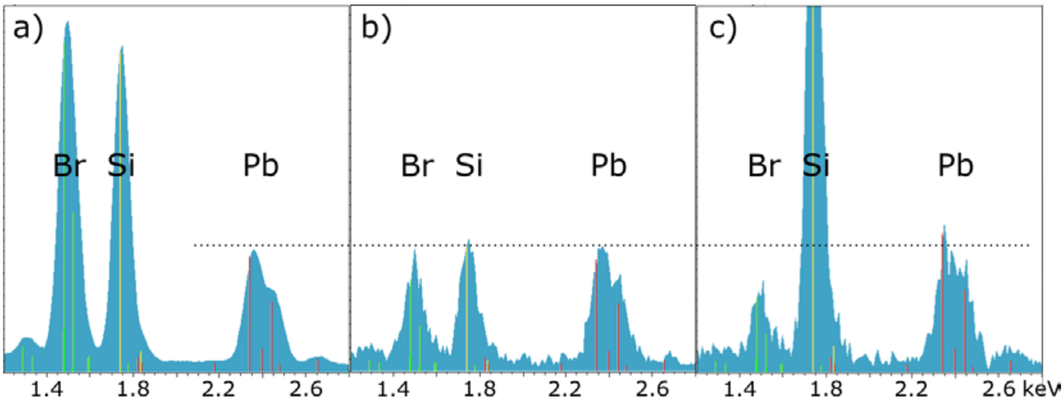

Supplementary Figure 2 EDS spetra of perovskite nanocrystals prepared with the precursor concentration of a) 2x, b) 1x and c) 0.33x, respectively. Vertical axis normalized to the height of Pb peak is indicated with dotted line.

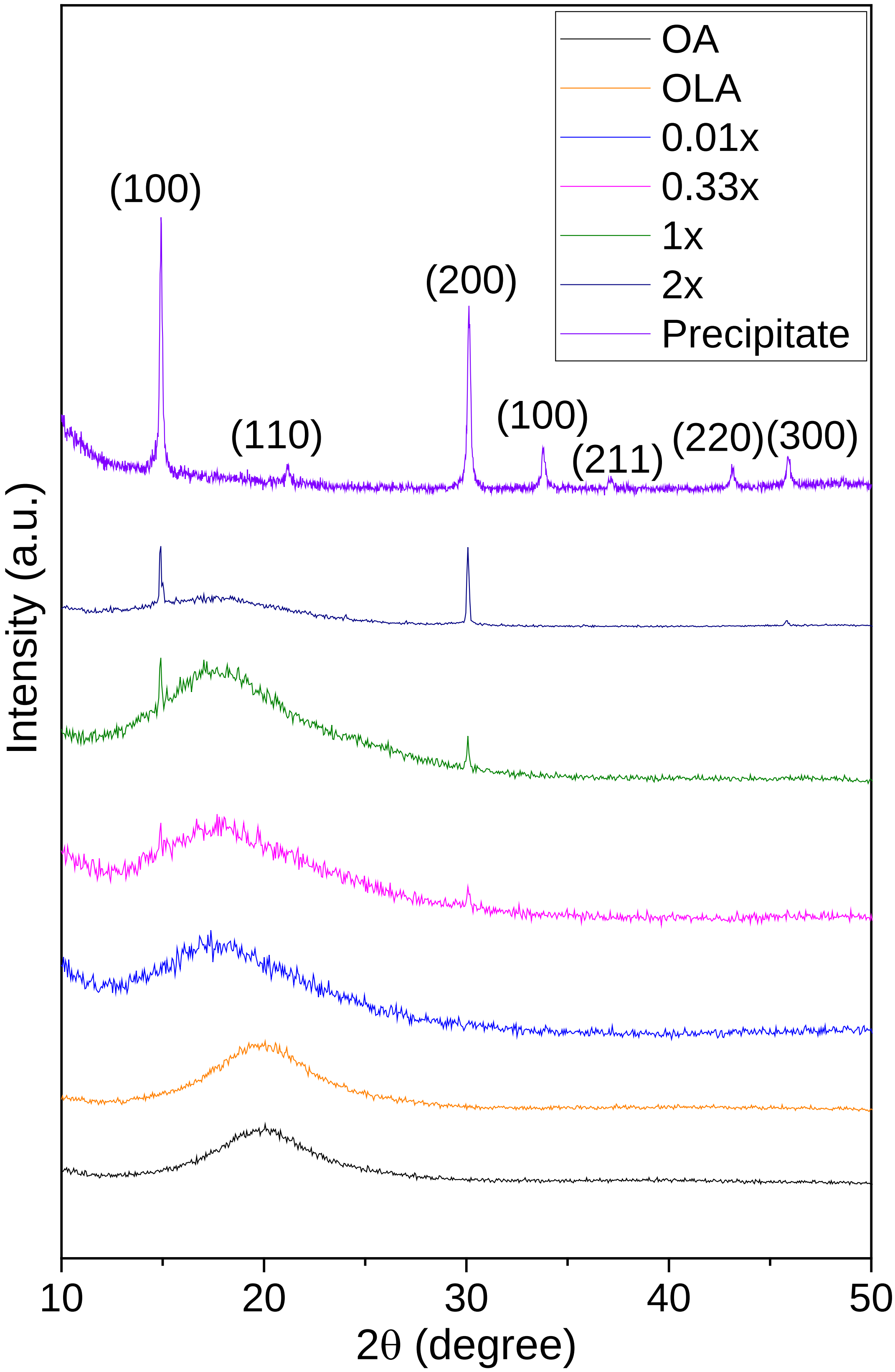

Supplementary Figure 3 XRD spectra of (from top to bottom; as indicated by different colors on the frame): OA and OLA ligands; perovskite nanocrystals prepared with the precursor concentration of 0.01x, 0.33x, 1x, and 2x respectively; and 2x perovskite precipitate.

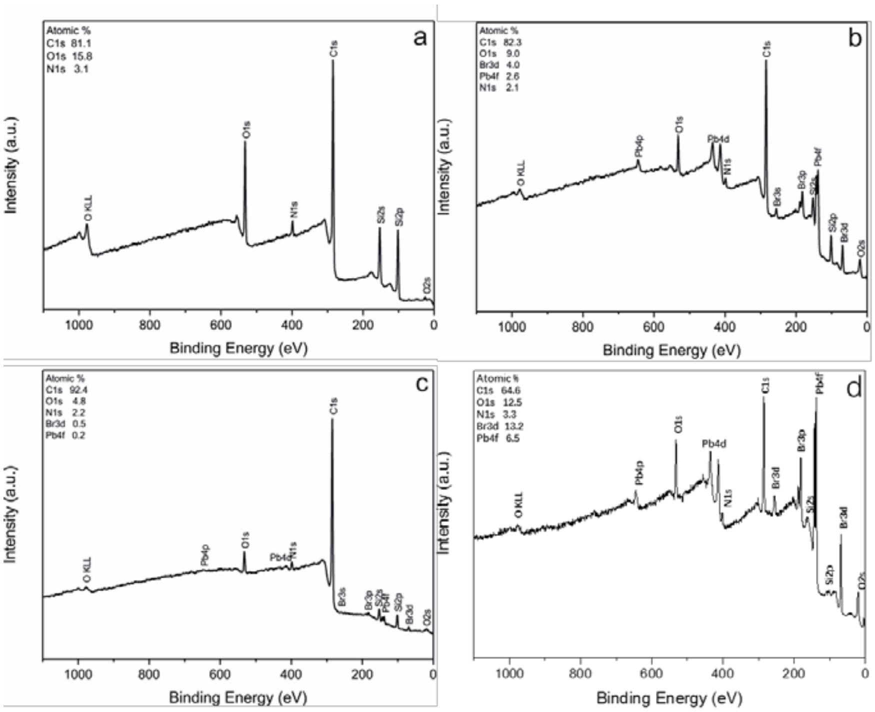

Supplementary Figure 4 XPS survey scans of perovskite nanocrystals prepared with the precursor concentration of 0.33x (a), 1x (b), 2x (c), and of the 2x perovskite precipitate (d), with relative quantifications.

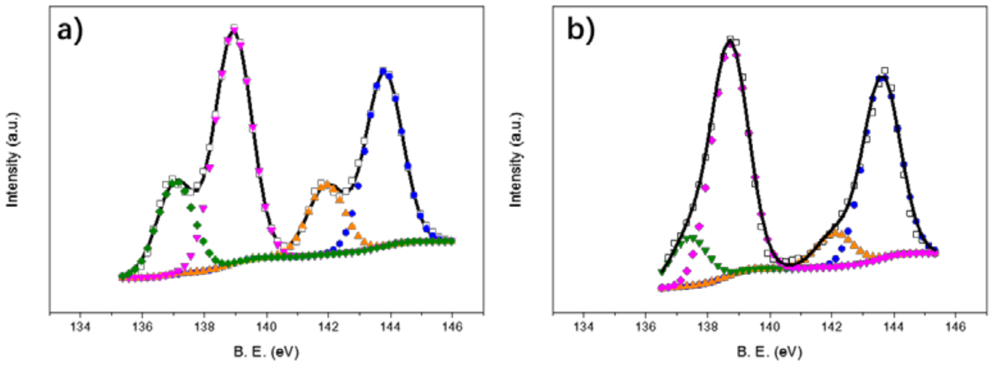

Supplementary Figure 5 XPS spectra (black dotted line – as measured; black solid line – the fit) of Pb 4f for perovskite nanocrystals prepared with precursor concentration of 1x (a), and 2x (b) respectively. Dotted lines show their deconvolution into 4 peaks.

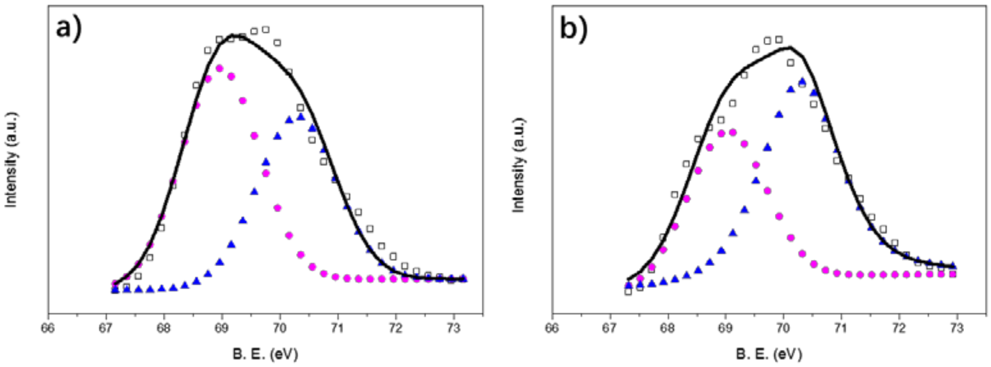

Supplementary Figure 6 XPS spectra (black dotted line – as measured; black solid line – the fit) of Br 3d for perovskite nanocrystals prepared with the precursor concentration of 1x (a), and 2x (b) respectively. Dotted lines show their deconvolution into 2 peaks.

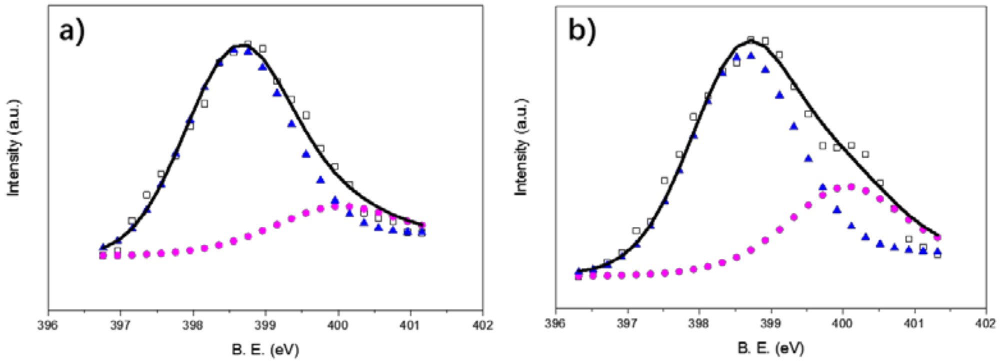

Supplementary Figure 7 XPS spectra (black dotted line – as measured; black solid line – the fit) of N 1s for perovskite nanocrystals prepared with the precursor concentration of 1x, and 2x, respectively. Dotted lines show their deconvolution into 2 peaks.

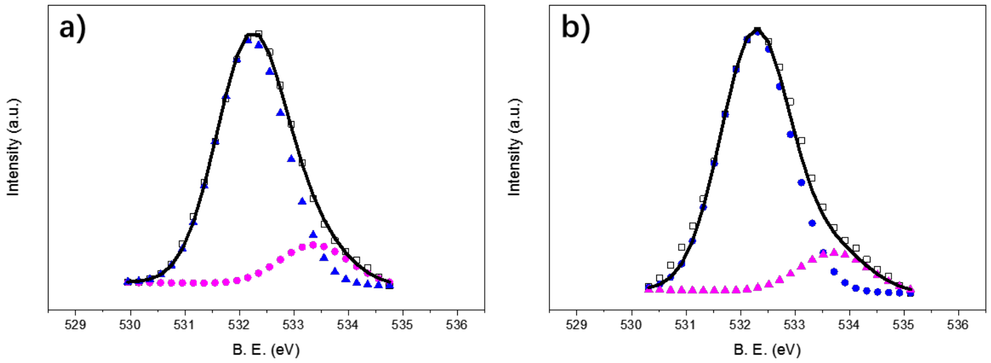

Supplementary Figure 8 XPS spectra (black dotted line – as measured; black solid line – the fit) of O 1s for perovskite nanocrystals prepared with the precursor concentration of 1x, and 2x, respectively. Dotted lines show their deconvolution into 2 peaks.

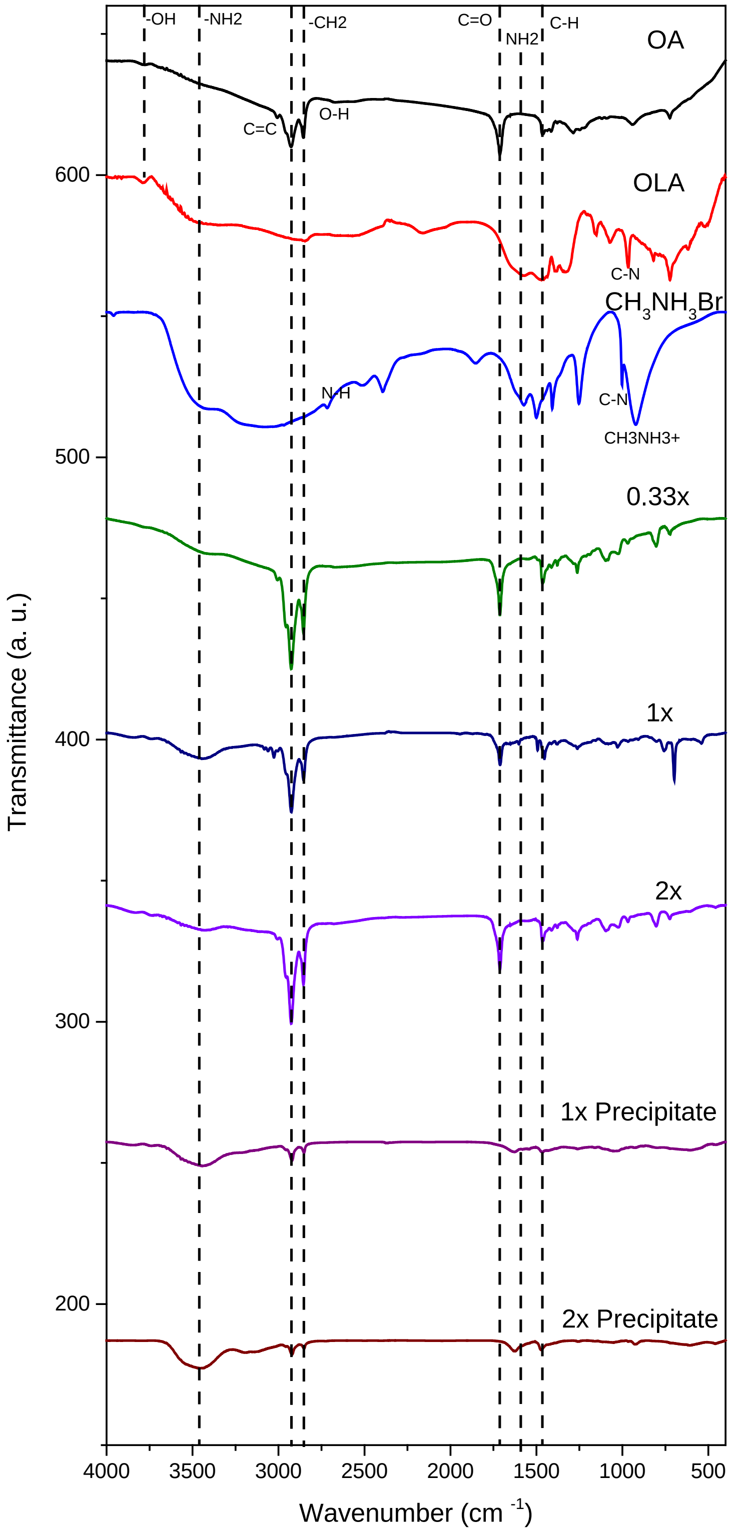

Supplementary Figure 9 FTIR spectra of (from top to bottom; as indicated by different colors on the frame): OA and OLA ligands, CH_3NH_3Br precursor, and perovskite nanocrystals prepared with the precursor concentration of 0.33x, 1x, and 2x respectively, as well of the 1x and 2x precipitates. Assignement to specific bands is given on the top of the frame.

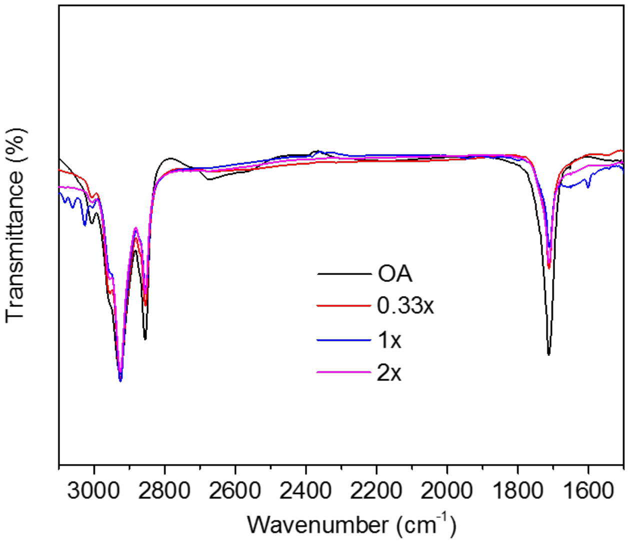

Supplementary Figure 10 FTIR spectra of (from top to bottom; as indicated by different colors on the frame): OA ligand and perovskite nanocrystals prepared with the precursor concentration of 0.33x, 1x, and 2x respectively, all normalized to the absorbance at 2925 cm^{-1}.
